# Psychometrics of the moral distress scale in Iranian mental health nurses

**DOI:** 10.1186/s12912-021-00674-4

**Published:** 2021-09-10

**Authors:** Raziyeh Ghafouri, Sara Lotfi-Bajestani, Malihe Nasiri, Kayoko Ohnishi, Foroozan Atashzadeh-Shoorideh

**Affiliations:** 1 Department of Medical-Surgical Nursing, School of Nursing & Midwifery, Shahid Beheshti University of Medical Sciences, Tehran, Iran; 2 Nursing Research Committee, School of Nursing & Midwifery, Shahid Beheshti University of Medical Sciences, Tehran, Iran; 3 Department of Basic Sciences, School of Nursing & Midwifery, Shahid Beheshti University of Medical Sciences, Tehran, Iran; 4 Faculty of Nursing and Rehabilitation, Konan Women’s University, Kobe, Hyogo Japan; 5 Department of Psychiatric Nursing and Management, School of Nursing and Midwifery, Shahid Labbafinezhad Hospital, Shahid Beheshti University of Medical Sciences, Tehran, Iran

**Keywords:** Moral distress, Mental health, Nurse, Psychometrics

## Abstract

**Background:**

One of the challenges that nurses often face in ethical decision-making situations is moral distress. Moral distress is caused by the conflict between professional and individual values in decision-making situations. Despite its importance, there is no reliable scale in Persian to measure it. Therefore, this study was conducted to validate the moral distress scale in mental health nurses in Iranian culture and Persian language.

**Methods:**

This study was conducted in two parts: Translation and cross-cultural adaptation and psychometric analysis. The translation and cross-cultural adaptation process was conducted based on the Polit approach. Next, face validity (qualitative), content validity (quantitative and qualitative), and construct validity were examined. This part of the study was a cross-sectional study. In this step, a demographic questionnaire and the Moral Distress Scale were sent to 500 nurses working in selected educational and medical centers in Iran via online questionnaires. Then, the construct validity of the “Moral Distress Scale” was confirmed by confirmatory factor analysis and the reliability of the instrument was examined by studying the internal consistency with Cronbach’s alpha and the internal correlation of the AIC.

**Results:**

The confirmatory factor analysis showed an acceptable ratio of the expressions in 15 items in three factors: Acquiescence to patients’ rights violations (6 items), Unethical conduct by caregivers (5 items), and low staffing (4 items) in the scale. The internal consistency of the instrument with Cronbach’s alpha was higher than 7.0.

**Conclusion:**

The Persian version of moral distress with 15 items of the three factors had validity and reliability. According to the present findings, this scale can be used to study moral distress among nurses working in psychiatric wards. Moral distress leads to burnout, increases risks to patient safety and reduces quality of care. Nurses need to be able to assess and manage moral distress. Therefore, considering the side effects, it is necessary to have a reliable and valid scale that can be studied. Considering that culture has an impact on nurses’ moral distress, it is suggested that this instrument be studied in and tested in other languages and cultures.

**Supplementary Information:**

The online version contains supplementary material available at 10.1186/s12912-021-00674-4.

## Background

Moral distress arises from the conflict between professional and individual values [1, 2] and is one of the challenges nurses face in many situations involving ethical decision making. Working with incompetent staff, unsafe working conditions, organizational constraints, and shortage of manpower and equipment are some of the problems that cause moral distress in nurses [3–5]. Moral distress occurs when a person knows what a good job is, but organizational constraints or fears about the consequences of the job make it almost impossible to do the right thing [5].In the United States, one in three nurses suffers from moral distress [6]. Approximately 5.17% of novice nurses leave their job in the first year due to moral distress [7].

The moral distress experienced by nurses in various situations [8], causes feelings of failure and guilt, anger, job dissatisfaction, stress, turnover, sadness, anxiety, feelings of shame, low self-esteem, feelings of burnout, feelings of insecurity, fear, discouragement, and depression in nurses and these consequences can affect their personal lives and professional performance [3–5, 8–10]. The psychological trauma of moral distress disrupts nurses ‘daily lives [1, 11] and causes nurses to leave both their jobs and the profession itself [12–14]. Therefore, understanding the concepts related to moral distress can play an important role for nurses and nurse managers in reducing moral distress, improving the quality of nursing services, and patient satisfaction [12, 15].

Moral distress not only has a negative impact on nurses, but also on the organization and patient care [3]. Moral distress can cause nurses to isolate themselves from patients, patients not to defend herself, and patients to become uncomfortable (effect of moral distress on the patient) [3, 12, 15].On the other hand, the effects of moral distress on nurses can take the form of resignation, burnout, and leaving the nursing profession [12, 14]. The negative effects of moral distress on organizations include increasing the number of nurses leaving the service, decreasing the number of experienced nurses and quality of nursing care, and the reputation of the organization [16]. Therefore, identifying moral distress and the factors behind it plays a vital role in management and future planning [17]. A valid and reliable cross-cultural scale would be beneficial to capture moral distress.

Moral Distress was defined in nursing in the 1980s [18], and Corley designed the scale with 36 items in 1995 [19] and modified it in 2005 [2] This modified scale showed good reliability and validity [14]. Considering that environmental and cultural factors influence moral distress and compliance with it [20], the perception of moral distress is different in different environments and cultures [8, 21] and moral distress differs according to the conditions and work environment [22], Corley’s scale was developed and psychometrically tested for critical care nurses [15] and therefore is not recommended for use in other settings.

Because moral distress varies by work setting, nurses in mental health care may experience a different type of moral distress than nurses in other settings because of restrictions on patient freedom (e.g., involuntary hospitalization, restraint). Little is known about the moral distress experienced by nurses in mental health care. Yet mental health wards are one of the environments with more moral distress [1]. The moral distress of nurses in mental health wards in caring for patients with severe mental illness at any stage of illness and with various conditions, including cases where the patient’s vision is impaired [16], is higher than in other treatment environments [1, 16]. They should also pay attention to families who suffer from caring for their patients and are often unknowingly exposed to internal and external constraints [16]. Therefore, specific scales are needed to study moral distress in mental health services.

For the first time, in 2010, Ohnishi et al. introduced the Scale for Measuring Moral Distress for Psychiatric nurses (MDS-P) to examine nurses’ experience of moral distress. Because the Moral Distress Scale (MDS) focuses primarily on moral distress about physical care, it is inappropriate for use in a mental health setting. The MDS-P uses some items from Corley’s 30-Phrase Moral Distress Scale, adding items applicable for mental health care [1]. It is a 15-item scale designed to include three subscales: “Unethical conduct by Caregivers” (six terms), “Low staffing” (Five phrases) and “Acquiescence to patients’ rights violations” (four cases) [1]. The MDS-P includes questions about long-term hospitalization and unethical behavior of caregivers, such as inadequate care, covertly mixing medications into patients’ food, ridiculing patients, searching for patients’ belongings, and handling shopping rather than letting patients go shopping [10]. The validity and reliability of this scale has been studied in Japan [1]. Due to the influence of environment on moral distress, further studies are needed in this field and the provided scales should be adapted to different cultures [20]. Considering the problems such as dissatisfaction and burnout that moral distress brings to nurses, it is necessary to have an appropriate instrument to assess it in nurses working in mental health wards. Despite the importance of the concept of moral distress among nurses working in mental health wards, there is no valid and reliable scale in this regard in Persian. A valid and reliable instrument to assess moral distress can be helpful in identifying the incidence of moral distress and developing prevention programs. Therefore, this study was conducted to validate the moral distress scale among mental health nurses in Iranian culture and Persian language.

## Methods

### Objectives

The aim of present study is to translate the “Moral distress Scale” and then use it to assess Iranian mental health nurses.

### Design

The present study was conducted by a cross-sectional descriptive method. It was conducted in two parts: translation and cross-cultural adaptation and Psychometric assessment. After obtaining permission from the original scale designer, the translation and cross-cultural adaptation process was performed based on the Polit approach [23]. In the next step, face validity (qualitative), content validity (quantitative and qualitative), and structural validity were examined.

### Cultural adaptation

Polit approach includes seven steps of translating the scale from English to Persian, combining the original translations, translating the final version from Persian to English reviewing the version translated from Persian to English, conducting a preliminary study to test the scale, modifying, summarizing and testing the translated questionnaire [23]. To perform transcultural translation, first, the questionnaire was translated from English to Persian by two experienced translators then both versions were compared and combined. The scale prepared in Persian were distributed among 10 nurses to eliminate ambiguities and any other problems. After the corrections, the Persian translation of the inventory was re-translated to English by two bilingual people. At this stage, the new English version was sent to the scale designer and approved after she made further corrections. In qualitative face validity, patients’ views on the level of difficulty, relevance, and ambiguity were examined and the necessary corrections were applied to better understand some expressions.

### Setting

The study population was made up of nurses working on the mental health wards of educational and medical centers affiliated to medical universities from different regions of Iran. Data was collected from May until August 2020.

### Participants

The sample size was estimated at 450 according to the number of items (30 samples per item) [24], and at 500 according to the sample loss. The link to the list was provided online to nurses in different hospitals of the country and 500 nurses completed the instrument.

#### Including criteria

Nurses who was work in mental health setting at least for 1 year and willing to participate the research.

#### Excluding criteria

Instrument that was not full completed, those who were not willing to respond and those nurses with less than 1 year of experience in mental health nursing. Generally, the adjustment or integration phase occurs after 6 to 12 months, depending on the individual, when routines develop and things feel normal.

### Instruments

Demographic questions included age, sex, and marital status, level of education, work experience and shift work. The second part of the questionnaire was 15 phrases of the MDS-P scale (supplementary file). MDS-P scale containing three sub-scales: “Unethical conduct by Caregivers” (Items 4, 1, 2, 13, 10 and 7), “Low staffing” (items 6, 9, 3, 15 and 12) and “Acquiescence to patients’ rights violations” (items 11, 8, 5 and 14) was designed [1].

### Data analysis

Content Validity Index (CVI) and Content Validity Ratio (CVR) were then calculated to assess the quantitative content validity. To assess content validity, 10 nurses were asked to provide comments on grammar, use of appropriate words, proper placement of phrases, and appropriate scoring. Ten nursing experts were asked to rate each item to rate the CVR using the following equation the following equation CVR=)n_e_-N/2)/(N/2) [25].

The CVR is between = 1 and − 1 and a score above zero indicates that an item is required. The minimum acceptable CVR score was checked using the Lawshe’s table and items with a score below the minimum acceptable level were removed [26]. The CVI was then calculated using the following formula, which is higher than 79.0.

After collecting the data, analysis was done on the completed questionnaires’ data. Construct validity was then assessed by Confirmatory Factor Analysis (CFA) to confirm three factor of scale mentioned previously by the scale’s designer. The model fit was assessed based on the Root Mean Square Error of Approximation (RMSEA), Comparative Fit Index (CFI), Normed Fit Index (NFI), Goodness of Fit Index (GFI), and Adjusted Goodness of Fit Index (AGFI). To judge the model fit, Comparative Fit Index (CFI), Standardized Root Mean Squared Residual (SRMSR), and Root Mean Squared Error of Approximation (RSMEA) were considered with cut points of > 0.95, < 0.06, and < 0.08, respectively [19].

The reliability of the instrument was assessed by two methods of internal consistency with Cronbach’s alpha and the average inter-item correlation (AIC). Cronbach’s alpha lower than 0.3 is considered low reliability, between 0.3–0.7 fair, and more than 0.7 is considered as good reliability [20, 21]. Analyses were conducted using IBM SPSS Statistics, Version 20 (IBM Corp., Armonk, NY) and LISREL (LInear Structural RELations) software version 8.80 (By Karl G. Joreskog & Dog Sorbom., Lincolnwood, IL 60712, USA).

## Results

### Characteristics of the study participants

The mean age of participants was 37.59 ± 7.03. Table 1 presents the demographic characteristics of the participants.

**Table 1:** Demographic characteristics of the participants

Variable	Range	Number	Percentage
**Age**	23–32 years	96	19.2
	33–42 years	260	52
	43–52 years	110	22
	52–63 years	13	2.6
	Missing data	21	4.2
**Sex**	Male	112	22.4
	Female	377	75.4
	Missing data	11	2.2
**Marriage**	Married	156	31.2
	Single	289	57.8
	Divorced	25	5.0
	Widow	14	2.8
	Lived Alone	6	1.2
	Missing data	10	2.0
**Education**	Bachelor	426	85.2
	Master	63	12.6
	Ph.D.	2	.4
	Missing data	9	1.8
**Work in psychology ward**	1–5 years	205	41
	5–15 years	250	50
	16–30 years	28	5.6
	> 30 years	5	1
	Missing data	12	2.4

### Content validity

In the qualitative content validity assess, the face validity was confirmed and items 2, 4, and 5 were modified and simplified with the opinion of experts. Next, the quantitative content validity assess, the CVR was higher than 0.49 and the CVI of all expressions was higher than 0.9.

### Construct validity by confirmatory factor analysis (CFA)

The fit of the model was confirmed using the chi-square test with *P* < 001. Confirmatory factor analysis showed an acceptable proportion in 15 items expressions in three factors: Acquiescence to patients’ rights violations (6 items), Unethical Conduct by Caregivers (5 items), and Low staffing (4 items) in the scale (Table 2).

**Table 2:** Rotated component Matrix for the Moral Distress Scale in Mental health nurses (Persian version)

No of Items	Items	Factor		
		1	2	3
a4	Assist the physician who does a test without patient permission and obtaining informed consent.	**0.73**	0.17	0.19
a1	Follow the family’s wishes for the patient’s care when the hospital management don’t agree with them, but do so because of fears of lawsuit.	**0.72**	0.23	0.26
a2	Follow the orders of the doctor, who prioritizes the preferences of the family over the patient.	**0.67**	0.29	0.26
a13	Hide meds in food and drink, when patients refuse to take them.	**0.65**	0.28	0.34
a7	Work with levels of healthcare staffing that I consider unsafe.	**0.64**	0.26	0.30
a10	Witness healthcare providers giving false hope or not telling the truth to a patient or family.	**0.63**	0.18	0.21
a6	Avoid taking action when I learn that a colleague has made a medical error and does not report it.	0.17	**0.71**	0.12
a9	Witness healthcare providers make fun of a patient and do nothing about it.	0.21	**0.68**	0.16
a3	Carry out medical orders for what I consider unnecessary tests and treatments.	0.19	**0.63**	0.25
a15	Work in an organization that not treating the nurses well.	0.16	**0.60**	0.33
a12	Provide less than optimal care to an unconscious patient because of being short-staffed.	0.33	**0.57**	0.08
a11	Witness and take no action for discharging of a patient that no longer needs to be there and is ready to get back to normal life.	0.24	0.23	**0.78**
a8	Provide less than optimal care due to pressures from administrators or insurers to reduce cost.	0.34	0.18	**0.56**
a5	Detecting and ignoring suspicious patient abuse and neglect by caregivers.	0.46	0.22	**0.53**
a14	Avoid talking to gentle and calm patients because of being overwhelmed at work.	0.27	0.26	**0.51**

Extraction Method: Maximum Likelihood

Rotation Method: Varimax with Kaiser Normalization

The model fit in this study was 87 Minimum Fit Function Chi-Square = 259.18 (*P* = 0.00) and NFI = 0.97 RMSEA = 0.063, (GFI) = 0.93, CFI = 0.98 which gave an acceptable fit of the model and items were approved in 3 factors (Table 3).

**Table 3:** Fitness of model results

	Chi-Square	df	***P*** value	RMSEA	CFI	NFI	GFI
**(3 factors)** **Model fit**	259.18	87	P < 001	0.063	0.98	0.97	0.93

**Fig. 1:**
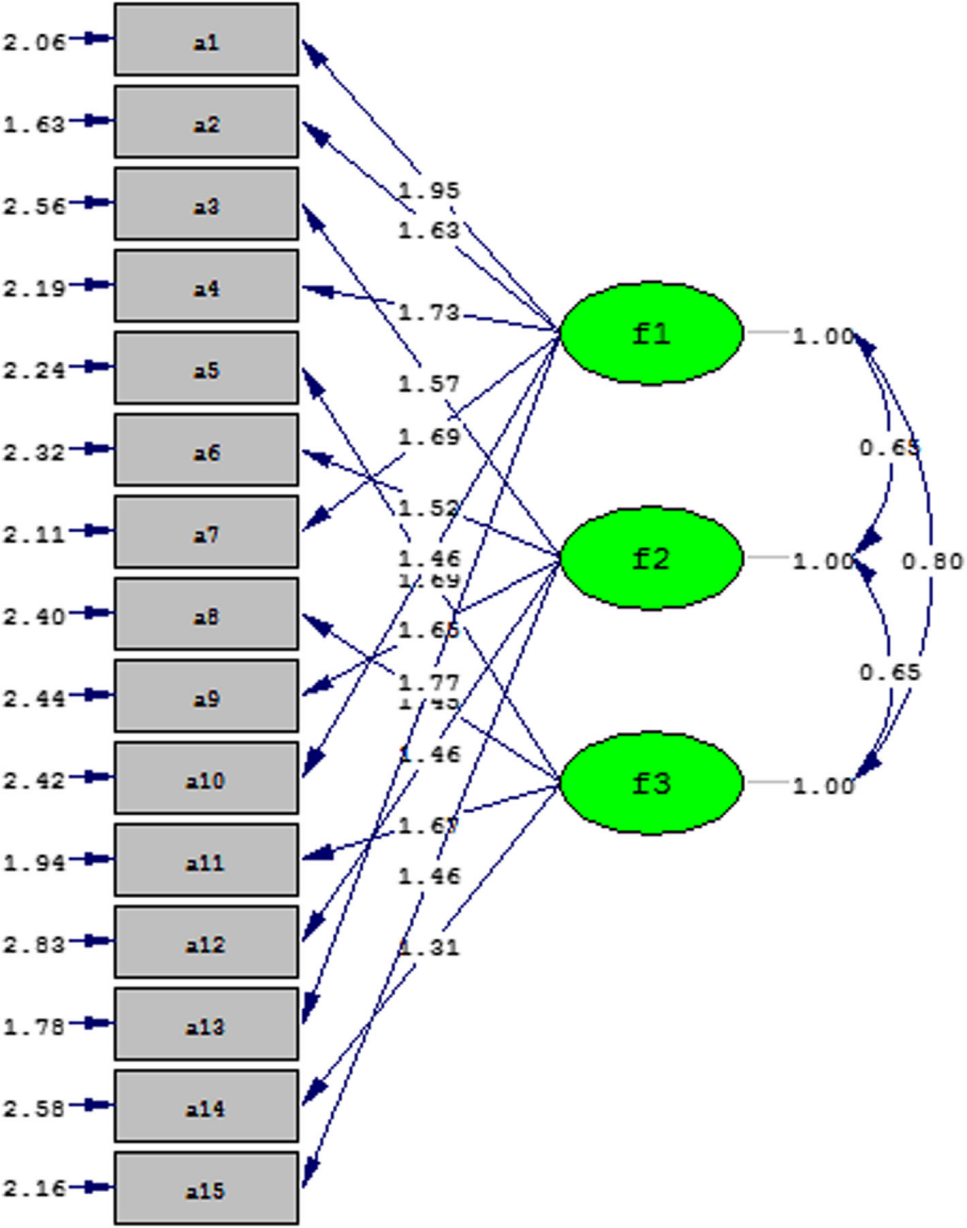
Model fitness

Items 4, 1, 2, 13, 7, and 10 in Acquiescence to patients’ rights violations, items 6, 9, 3, 15, 12 in Unethical Conduct by Caregivers and other items (11, 8, 5, and 14) were in the Low staffing factor (Fig. 1).

### Reliability

Cronbach’s alpha was 0.916. In Acquiescence to patients’ rights violations α = 0.896, AIC: 0.65, in Unethical conduct by caregivers α = 0.82, AIC: 0.62 and in Low staffing factor α = 0.80, AIC: 0.62 and it showed that AIC and Cronbach’s alpha was also in the approved range in the agents and the reliability of the instrument was appropriate.

## Discussion

The translation and cultural adaptation of the scale is done to better adapt the scale to the culture and language of the target community to better understand the items and respond to them [23, 27, 28]. After translation and back translation, validity and reliability are the key indicators in each scale [23, 27] which are confirmed in different ways. In this study, face validity was confirmed by quantitative and qualitative methods, and content validity of the translated scale was confirmed. Items 4, 1, 2, 13, 10, and 7 in the factor unethical Conduct by Caregivers, items 6, 9, 3, 15, 12 in the factor Low Staffing and other items 11, 8, 5, 14 were included in the factor Acquiescence to patients’ rights violations, which is consistent with the results of Ohnishi et al. [1].

If moral distress is not detected, it can lead to burnout and decreased patient safety and quality of care. Moral distress not only has a negative impact on the nurse, but also has negative consequences in patient care. Nurses must be able to cope with moral distress, make the right decision, and provide quality patient care. Therefore, considering the side effects, it is necessary to have a reliable and valid scale that can be studied. The results of this study showed that the Persian version of moral distress with 15 items of the three factors has validity and reliability. Similar studies that investigated this scale in different communities, were not found. Therefore, a comparison of the factors of MDS Corley’s and its studies in certain sections was used in the discussion.

The 36-item MDS Corley scale examines moral distress in nurses working in intensive care units [11, 29]. In the study of Shoorideh et al., who studied the moral distress in nurses working in intensive care unit in Iran using the Corley scale, three factors were identified: “inappropriate competencies and responsibilities”, “errors”, and “not respecting the ethics principles” [5]. Similarly, Soleimani et al. studied the scale of moral distress in the intensive care units and identified 5 factors for it, including the role of the health care provider, futile care, obedience to the physician, lack of trust of the patient, and limitation of the organization [30] which had different results from the results of Corley’s research. The results of the above research also emphasize the neglect of patients’ rights as well as the occurrence of errors. This finding was highlighted in the present study and the results of the research by Ohnishi et al. [1]. Therefore, it is necessary to familiarize nurses with educational decisions that ignore patients’ rights in educational programs.

The first factor from the current study is acquiescence to patients’ rights violations, which was also confirmed in the study by Ohnishi et al. [1]. Acquiescence to patients’ rights violations has also been emphasized in other studies such as Corley [2], Shoorideh et al. [5] and Soleimani et al. [30] in Iran, which shows that this factor causes moral distress when the nurse does not have the responsibility or the ability and confidence to support their patient and defend their rights.

Depending on the type of clinical area, different issues cause moral stress among nurses, and some issues are more important in mental health settings than in other units. It should be noted that in mental health settings, due to the patient being disoriented and being treated without his consent, there is a possibility of violation of his rights by his family or other caregivers. This can cause moral distress to those who witness these cases. Legal guidance in these cases, allowing nurses to feel more empowered to protect patients may help reduce the incidence of these cases. In the study by Ohnishi et al. the instrument was created according to the situations of moral distress prevalent in mental health settings [1]. Despite the confirmation of the validity of the scale by Ohnishi et al., studies on the application of the scales in different cultures seem necessary, as culture has a significant influence on values and ethical decisions and the occurrence of moral distress.

The next factor identified in the present study is the unethical conduct by caregivers which shows that the nurses do not have the necessary commitment to ethics. In the study of Shoorideh et al. the factor “not respecting the ethics principles” was identified, which emphasizes the observance of ethical principles by care providers [5]. Therefore, according to the results of the present study, which is in line with those of Ohnishi et al. [1] and has been studied in mental health wards, as well as the research of Shoorideh et al. [5], there seems to be a need to develop ethical commitment among care providers. This is to prevent unethical behaviors and the challenges associated with them, such as moral distress in and around the person and harm to the patient. This factor is also one of the important issues to be considered in this study. The development of ethical principles in nursing is a necessity. By developing ethical standpoint it is possible to prevent and address these cases. The best suggestion for developing ethical behavior is to introduce nurses to ethics as a nursing model.

The third factor identified in the present study, which is consistent with the study of Ohnishi et al. [1], is the low number of employees due to the shortage of staff and related challenges such as relying on less experienced staff. In the study of Shoorideh, inappropriate competencies and responsibilities were identified [5] and in the study of Soleimani et al. organizational factors were identified, one of the issues was the lack of qualified professionals [30]. The shortage of manpower and employment of the low skilled employees is influenced by organizational factors such as the cost of employment in the organization. It is a factor seen in different departments and nursing managers should take necessary steps to solve this problem.

Professional competence and scientific understanding and skill development among nurses and other care providers can help reduce the incidence of ethical distress. Assessing the skills and knowledge of nurses when they are first employed on a particular ward and periodically thereafter is one of the issues that is always emphasized. Unfortunately, due to manpower shortage, sometimes nurses are hired with low skills or little experience. In cases where nurses are guided by their values and ethical principles, the lack of sufficient knowledge and skills leads to unwanted mistakes, and unwanted errors cause severe moral distress and burnout in these individuals, which leads to leaving the job. Therefore, in order to improve the quality of care and prevent the occurrence of moral distress and burnout, it is necessary for nurses to have access to continuous professional development so that knowledge and skills can be evaluated and enhanced.

In the study of Ohnishi et al. KMO method was used to investigate the exploratory factor analysis and identify the factors, indicating the accuracy of the above study, and then the identified factors were confirmed by the confirmatory factor analysis. In the present study, the factors were confirmed by the confirmatory factor analysis. In the study of Ohnishi et al. Cronbach’s alpha was 0.80 for unethical behavior by caregivers, 0.75 for low staffing, 0.72 for patient rights compliance, and 0.89 for the whole scale [1]. The results of the reliability study of the instrument in Iran were also confirmed and showed that the instrument has the necessary validity and reliability. Despite the confirmation of the validity and reliability of Ohnishi et al.’S morality scale in Iran, it is necessary to conduct studies in other settings and cultures due to the influence of culture and conditions in mental health settings.

Based on the results of the present study, the Persian version of moral distress with 15 items and 3 validity and reliability factors is suitable for use in the study of moral distress in mental health wards. Given the various cultural factors impact on moral distress, [20] further studies should be conducted to validate and transcultural translation in different cultures.

### Limitations and strengths

One of strengths of this research was the sufficient number of participants from different educational and medical centers of Iran. Limitations of this study include the lack of a Persian instrument similar to the MDS to assess the concurrent validity. Another limitation of this study was that discriminant validity, convergent validity, and test-retest reliability were not used. Therefore, this investigation is highly recommended in the future researches. Discussion about findings of this study is difficult, because there are not similar studies about translation and validation of MDS-P in other cultures and languages.

## Conclusion

If moral distress is not detected, it can lead to burnout, decreased patient safety, and quality of care. Moral distress not only has negative effects on the nurse, but also has negative consequences in patient care. Nurses must be able to assess and deal with moral distress, make the right decision, and provide quality patient care. Therefore, considering the side effects, it is necessary to have a reliable and valid scale that can be studied. The results of this study showed that the Persian version of moral distress with 15 items of the three factors has validity and reliability. According to the related findings, this scale can be used to study moral distress among nurses working in mental health wards.

## Supplementary Information

**Additional file 1.**

## Data Availability

All data generated or analysed during this study are included in this published article and its supplementary file (Moral Distress for Psychiatric nurses).

## Abbreviations

MDS-P: Moral Distress for Psychiatric nurses
CVR: Content validity ratio
CVI: Content validity index
KMO: Kaiser-Meyer-Olkin
MLE: Maximum-Likelihood Estimation
RMSEA: Root Mean Square Error of Approximation
CFI: Comparative Fit Index
NFI: Normed Fit Index
GFI: Goodness of Fit Index
AGFI: Adjusted Goodness of Fit Index
AIC: Average Inter-item Correlation

## References

[1] Ohnishi K, Ohgushi Y, Nakano M, Fujii H, Tanaka H, Kitaoka K, Nakahara J, Narita Y (2010). Moral distress experienced by psychiatric nurses in Japan. Nurs Ethics.

[2] Corley MC, Minick P, Elswick RK, Jacobs M (2005). Nurse moral distress and ethical work environment. Nurs Ethics.

[3] Haghighinezhad G, Atashzadeh-Shoorideh F, Ashktorab T, Mohtashami J, Barkhordari-Sharifabad M (2019). Relationship between perceived organizational justice and moral distress in intensive care unit nurses. Nurs Ethics.

[4] Robaee N (2018). Perceived organizational support and moral distress among nurses. BMC Nurs.

[5] Shoorideh FA, Ashktorab T, Yaghmaei F, Alavi Majd H (2015). Relationship between ICU nurses’ moral distress with burnout and anticipated turnover. Nurs Ethics.

[6] Berhie AY, Tezera ZB, Azagew AW (2020). Moral distress and its associated factors among nurses in Northwest Amhara regional state referral hospitals, Northwest Ethiopia. Psychol Res Behav Manag.

[7] Krautscheid L, Mood L, McLennon SM, Mossman TC, Wagner M, Wode J (2020). Examining relationships between resilience protective factors and moral distress among nursing students. Nurs Educ Perspect.

[8] Prentice T, Janvier A, Gillam L, Davis PG (2016). Moral distress within neonatal and paediatric intensive care units: a systematic review. Arch Dis Child.

[9] Pauly BM, Varcoe C, Storch J. Framing the issues: moral distress in health care. Hec forum. 2012;24(1):1-11. 10.1007/s10730-012-9176-y. 10.1007/s10730-012-9176-y PMC3348467 22446885

[10] Shorideh FA, Ashktorab T, Yaghmaei F (2012). Iranian intensive care unit nurses’ moral distress: a content analysis. Nurs Ethics.

[11] Elpern EH, Covert B, Kleinpell R (2005). Moral distress of staff nurses in a medical intensive care unit. Am J Crit Care.

[12] Lievrouw A, Vanheule S, Deveugele M, Vos M, Pattyn P, Belle V, Benoit D (2016). Coping with moral distress in oncology practice: nurse and physician strategies. Oncol Nurs Forum.

[13] Oh Y, Gastmans C (2015). Moral distress experienced by nurses: a quantitative literature review. Nurs Ethics.

[14] Hamric AB, Borchers CT, Epstein EG (2012). Development and testing of an instrument to measure moral distress in healthcare professionals. AJOB Prim Res.

[15] Lamiani G, Borghi L, Argentero P (2017). When healthcare professionals cannot do the right thing: a systematic review of moral distress and its correlates. J Health Psychol.

[16] Wilson MA (2018). Analysis and evaluation of the moral distress theory. Nursing forum.

[17] Lachman VD (2016). Moral resilience: managing and preventing moral distress and moral residue. Med Surg Nurs.

[18] Corley MC (1995). Moral distress of critical care nurses. Am J Crit Care.

[19] Corley MC, Elswick RK, Gorman M, Clor T (2001). Development and evaluation of a moral distress scale. J Adv Nurs.

[20] Hamric AB (2012). Empirical research on moral distress: issues, challenges, and opportunities. HEC forum.

[21] Sannino P, Giannì ML, Re LG, Lusignani M (2015). Moral distress in the neonatal intensive care unit: an Italian study. J Perinatol.

[22] Hamaideh SH (2014). Moral distress and its correlates among mental health nurses in Jordan. Int J Ment Health Nurs.

[23] Polit DF, Yang FM. Measurement and the measurement of change: a primer for the health professions. 2015: Wolters Kluwer.

[24] Stevens JP (2012). Applied multivariate statistics for the social sciences.

[25] Lawshe CH (1975). A quantitative approach to content validity. Pers Psychol.

[26] Ayre C, Scally AJ (2014). Critical values for Lawshe’s content validity ratio: revisiting the original methods of calculation. Meas Eval Couns Dev.

[27] Polit DF, Beck CT (2006). The content validity index: are you sure you know what's being reported? Critique and recommendations. Res Nurs Health.

[28] Af Sandeberg M (2017). To change or not to change-translating and culturally adapting the paediatric version of the moral distress scale-revised (MDS-R). BMC Med Ethics.

[29] Morley G, Ives J, Bradbury-Jones C, Irvine F (2019). What is ‘moral distress’? A narrative synthesis of the literature. Nurs Ethics.

[30] Soleimani MA, Sharif SP, Yaghoobzadeh A, Panarello B (2019). Psychometric evaluation of the moral distress scale–revised among Iranian nurses. Nurs Ethics.

