# Supplementary material for: Psychometrics of the moral distress scale in Iranian mental health nurses
Source: BMC Nurs. 2021 Sep 10;20:166. doi: 10.1186/s12912-021-00674-4 (PMC8431900; doi:10.1186/s12912-021-00674-4)
Supplement: Supplementary file 1 — Additional file 1. [file 12912_2021_674_MOESM1_ESM.docx]

| **Moral Distress Scale for Psychiatric nurses- Persian version** |
| --- |
| 1. Follow the family’s wishes for the patient’s care when the hospital management don’t agree with them, but do so because of fears of lawsuit. |
| 1. Follow the orders of the doctor, who prioritizes the preferences of the family over the patient. |
| 1. Carry out medical orders for what I consider unnecessary tests and treatments. |
| 1. Assist the physician who does a test without patient permission and obtaining informed consent. |
| 1. Detecting and ignoring suspicious patient abuse and neglect by caregivers. |
| 1. Avoid taking action when I learn that a colleague has made a medical error and does not report it. |
| 1. Work with levels of healthcare staffing that I consider unsafe. |
| 1. Provide less than optimal care due to pressures from administrators or insurers to reduce cost. |
| 1. Witness healthcare providers make fun of a patient and do nothing about it. |
| 1. Witness healthcare providers giving false hope or not telling the truth to a patient or family. |
| 1. Witness and take no action for discharging of a patient that no longer needs to be there and is ready to get back to normal life. |
| 1. Provide less than optimal care to an unconscious patient because of being short-staffed. |
| 1. Hiding meds in food and drink when patients refuse to take them. |
| 1. Avoid talking to gentle and calm patients because of being overwhelmed at work. |
| 1. Work in an organization that not treating the nurses well. |
